# Synergistic Anticancer Effect of Paclitaxel and Noscapine on Human Prostate Cancer Cell Lines

**Published:** 2017

**Authors:** Arezou Rabzia, Mozafar Khazaei, Zahra Rashidi, Mohammad Rasoul Khazaei

**Affiliations:** *Fertility and Infertility Research Center, Kermanshah University of Medical Sciences, Kermanshah, Iran.*

**Keywords:** Apoptosis, Chemotherapeutic drugs, Noscapine, Paclitaxel, Prostate cancer

## Abstract

Paclitaxel is one of the most common chemotherapeutic drugs used for the treatment of prostate cancer. However, its current clinical utility has been limited due to numerous serious side effects and drug resistance. Noscapine is an antitussive opium alkaloid that showed antitumor activity against a variety of cancer while it has not exhibited severe side effects.

This study investigates the anticancer activity of noscapine in combination with paclitaxel against two LNCaP and PC-3 human prostate cancer cell lines*.* LNCaP and PC-3 cells were treated with noscapine or paclitaxel or combination. Cell viability was determined using 3-(4, 5-dimethylthiazol-2-yl)-2, 5-diphenyltetrazolium bromide (MTT) test. Apoptosis was assessed by acridine orange/ ethidium bromide (AO/EB) staining. The mRNA expression of *Bax*, *Bcl-2*, *AR,* and *PSA* in the cellular response to treatments was investigated. MTT assay indicated the combination treatment of paclitaxel and noscapine significantly decreased viability compared to single-agent treatment and control groups. The results obtained with AO/EB double staining showed increased percentages of apoptotic cells in the presence of the combination of paclitaxel and noscapine. Furthermore, combinational treatment of paclitaxel and noscapine showed significant decrease in the mRNA expression of B-cell CLL/Lymphoma (*Bcl-2*) and increase in the mRNA expression of Bcl-2-associated X protein (*Bax*(*,* and *Bax*/*Bcl-2* ratio in LNCaP and PC-3 cells (*P*<0.05.( The mRNA expression of androgen receptor* (AR*) and prostate specific antigen (*PSA*) decreased in paclitaxel and noscapine combination-treated of LNCaP cells (*P*<0.05). This study provides a novel concept of combination treatment of paclitaxel and noscapine to improve efficiency in human prostate cancer treatment.

## Introduction

Prostate cancer is the most common malignancy and the second leading cause of cancer- related death of men in the United States (1). The treatment options for patients include surgery, chemotherapy, radiation, and hormonal therapy, and combinations of some of these treatments. One of the most common chemotherapeutic drugs used for the treatment of prostate cancer are the taxanes (paclitaxel, taxol) (2, 3). Paclitaxel was derived originally from the Pacific Yew tree Taxus brevifolia (4) and it is current treatment of head and neck (5) , breast, ovarian, lung, prostate (2, 6), and gastric (7) cancers.

Paclitaxel is generally accepted to induce cell cycle arrest and apoptosis in most types of cancer cells ^(^8, 9). Paclitaxel interferes with microtubule assembly by binding and stabilizing β-tubulin in the G2/M phase of the cell cycle ^(^10, 11). However, clinical utility of paclitaxel has been limited due to numerous serious side effects (8, 21) and drug resistance (13). Noscapine is an antitussive opium alkaloid that lacks analgesic, euphoric, sedative, and respiratory depressant properties (14). Noscapine binds to tubulin and alters its conformation and assembly properties, interferes with microtubule dynamics both* in-vivo *and *in-vitro,* arrests a variety of mammalian cells in mitosis and targets them for apoptosis (14, 15, 16). However, noscapine is unable to compete with paclitaxel for binding to tubulin, suggesting that noscapine binds to a site other than the paclitaxel-binding site (17). Noscapine has showed antitumor activity against a variety of cancer: breast (14), melanoma (15), ovarian (17), gliobastoma (18), lymphoma (19), human myelogenous leukemia (20), and lung both *in-vitro* and *in-vivo* (21) while has not exhibited severe side effects that are commonly seen with many chemotherapeutic agents. Combination treatment with multiple therapeutic agents has been used to enhance the efficacy of treatment in controlling cancer cells (22, 23). In the present study, we investigated the synergistic anticancer activity of paclitaxel and noscapine against LNCaP and PC-3 human prostate cancer cell lines.

## Experimental

*Cell lines & drugs*

Human prostate cancer cell lines LNCaP (androgen receptor-positive; androgen-dependent) and PC-3 (androgen receptor-negative; androgen-resistant) were obtained from the National Cell bank of Iran (NCBI) and cultured in RPMI 1640 medium supplemented by 10% fetal bovine serum (FBS), penicillin G (100 IU/mL) and streptomycin (100 µg/mL). The cells were maintained at 37 °C in a humidified atmosphere in the presence of 5% CO_2_. All studies were done with cells at 70 to 80% confluence. Paclitaxel and noscapine were purchased from Sigma-Aldrich Co., USA .The drugs were dissolved in dimethyl sulfoxide (DMSO) and stored at −20 °C as a stock solution. The final concentration of DMSO in culture medium was 0.1% which was always nontoxic.

*Cell treatment*

LNCaP and PC-3 cell lines were seeded in 96-well plates, at a density of 2×10^4^ cells/well. After 24 h the cells were treated with various dilutions of paclitaxel (10, 25, 50 and 100 nM) or noscapine (10, 25, 50 and 100 μM) from stock solutions in DMSO for 24, 48, and 72 h.

In another set of experiments, the cells were treated with 50 nM Paclitaxel in combination with different concentrations of noscapine (10, 25, 50 and 100 μM) for 48 h, and untreated cells were served as control.

*Cell viability assay*

LNCaP and PC-3 cells viability was assessed by Cell proliferation kit I (MTT[3-(4,5-dimethylthiazol-2-yl)-2, 5-diphenyltetrazolium bromide]) Roche, GmbH, Germany) according to the manufacturer’s protocol. Briefly, 2×10^4 ^cells were plated in 96-well plates and exposed to serial concentrations of paclitaxel (10, 25, 50 and 100 nM) or noscapine (10, 25, 50 and 100 μM) for 24, 48, and 72 h. A separate study was done to find out the growth inhibitory effects of various concentration of noscapine (10, 25, 50 and 100 μM) on IC_50 _value (50 nM) of paclitaxel on LNCaP and PC-3 cells after 48 h. Each experiment was carried out at least in triplicate. All experiments were done at least in triplicate wells and the assays were repeated independently three times. 

The viability assay is based on the cleavage of yellow tetrazolium salt to purple formazan crystals by metabolic active cells (24). After the treatment periods, 10 µL of yellow MTT solution (final concentration 0.5 mg/mL) was added to each well and incbated at 37 °C in 5% CO_2_ for 4 h the purple formazan salt crystals were dissolved by adding solubilization solution. The absorbance of the samples was measured at 570 nm using a microplate (ELISA) reader (Stat fax 2100, USA). Cell viability calculated as the ratio of absorbance of treated groups divided into the absorbance of control group, multiplied by 100 to achieve a viability percent.

*Cell death assay*

LNCaP and PC-3 cells were treated with 50 nM paclitaxel, or 50 μM noscapine or combination for 48 h, and untreated cells were used as control. Treatments-induced apoptosis was carried by AO/EB (acridine orange/ ethidium bromide) double staining assay as previously described (25). Acridine orange is taken up by both viable and nonviable cells and emits green fluorescence if interrelated into double stranded nucleic acid (DNA). Ethidium bromide is taken up only by nonviable cells and emits red fluorescence by intercalation into DNA. We distinguished four types of cells according to the fluorescence emission and the morphological aspect of chromatin condensation in the stained nuclei. 

Viable cells have uniform bright green nuclei with organized structure. Early apoptotic cells (which still have intact membranes but have started to undergo DNA cleavage) have green nuclei, but perinuclear chromatin condensation is visible as bright green patches or fragments. Late apoptotic cells have orange to red nuclei with condensed or fragmented chromatin. Necrotic cells have a uniformly orange to red nuclei with condensed structure. The amount of 100 µL of dye mixture (100 µg/mL AO and 100 µg/mL EB in PBS) was added to cells in 96-well plate. The cells were viewed under a fluorescence microscope (Olympus, IX71). A minimum of 200 cells were counted in each sample. 

*RNA isolation and RT-PCR (reverse transcription polymerase chain reaction)*

LNCaP and PC-3 cells were treated with 50 nM paclitaxel or 50 μM noscapine and combination for 48. Untreated cells were used as control. The mRNA expression of *Bax *and *Bcl-2* was determined by Relative RT-PCR. The cells were harvested by trypsinization, washed three times, and pelleted by centrifugation. Total RNA was extracted using RNA purification kit (Jena Bioscience, GmbH, Germany). cDNA synthesis was performed by 0.5 μg of total RNA with AccuPower^®^ RocketScript^TM ^RT PreMix kit (Bioneer, Korea) with oligo dT and random hexamer primers in a volume of 20 µL. PCR was performed using oligonucleotide specific primers for *Bax*, *Bcl-2*, *AR*, P*SA.* *GA*P*DH *(glyceraldehyde-3-phosphate dehydrogenase) was used as an internal control. Amplification was performed by using the following primers: 

*Bax* forward primer 5´-GGGGACGAACTGGACAGTAA-3´, Reverse primer 5´-CAGTTGAAGTTGCCGTCAGA-3´ (GenBank accession no. NM_004324.3); 

*Bcl-2* forward primer 5´-ATGTGTGTGGAGAGCGTCAA-3´, reverse primer 5´ -ACAGTTCCACAAAGGCATCC-3´(GenBank accession no. NM_000633.2 (;

*AR *forward primer 5´- CCTGGCTTCCGCAACTTACAC-3´, reverse primer 5´- GGACTTGTGCATGCGGTACTCA-3´(GenBank accession no. NM_001011645.2); 

P*SA *forward primer 5´- ACCAGAGGAGTTCTTGACCCCAAA-3´, reverse primer 5´- CCCCAGAATCACCCGAGCAG-3´ (GenBank accession no. NM_001648.2). 

*GA*P*DH* forward primer 5´-CAGCCTCAAGATCATCAGCA-3´, reverse primer 5´- TGTGGTCATGAGTCCTTCCA-3´ (GenBank accession no. NM_002046.4). 

PCR reactions were carried out in total volumes of 20 µL using PCR PreMix kit (Bioneer, Korea) according to the manufacturer’s instructions with an AG PCR system (Eppendorf, Hamburg, Germany). The cycling conditions were as follows: initial denaturation at 94 ºC for 10 min, followed by 30 cycles of denaturation at 94 ºC for 1 min, 59 ºC (*GA*P*DH*), 60 ºC (*Bax* and *Bcl-2*), 66 ºC (P*SA*), 67 ºC (*AR*) annealing for 1 min and extension at 72 ºC for 1 min, with a final extension at 72 ºC for 10 min, following a 4 °C incubation for 10 min. 

*Relative RT-PCR analysis*

The mRNA expression of *Bax, *Bcl*-2,* P*SA,* and *AR* was quantified against the housekeeping gene (*GA*P*DH*). The PCR products were visualized by electrophoresis on 1.5% agarose gels followed by ethidium bromide staining (1 µg/mL) and photographed on an ultraviolet transilluminator (UVIdoc; Uvitec, Cambridge, UK). Gel images were analyzed using the Uvitec program. Relative RT–PCR values were presented as a ratio of the density of bands divided by density of *GA*P*DH* bands. Each experiment was repeated at least three times. 

*Statistical analysis*

Results were obtained through at least three separate experiments. One-way ANOVA test was performed to determine the significance of differences among groups using SPSS statistical software (version 16.0 SPSS Inc.). All data are shown as means ± SEM. and values of *P*<0.05 were considered significant.

## Results

*Effects of paclitaxel and noscapine on LNCaP and PC-3 cells viability*

**Figure 1:**
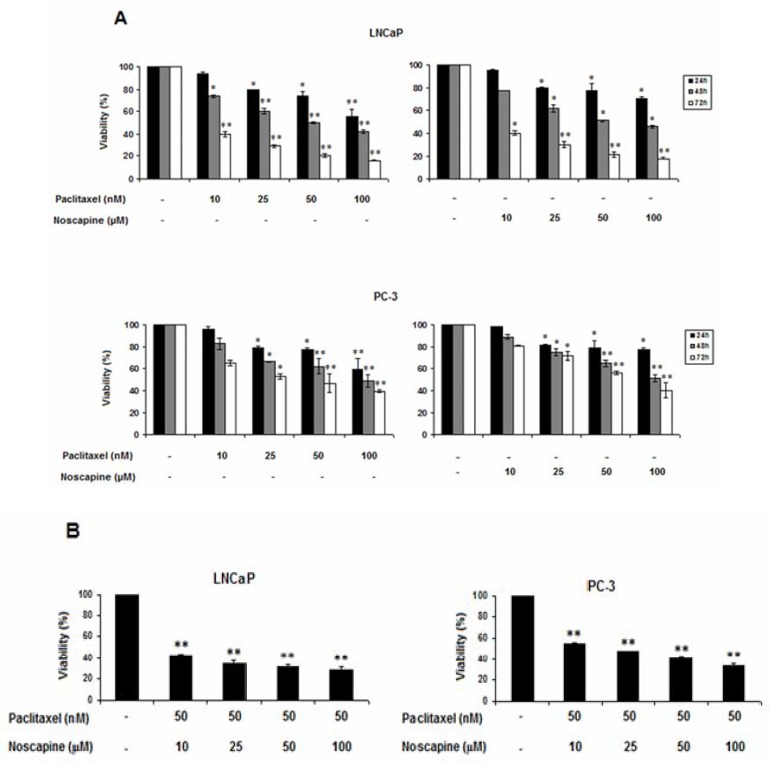
Effects of paclitaxel and noscapine on LNCaP and PC-3 cells viability using MTT assay. A: Effect of different concentrations of paclitaxel and noscapine on LNCaP and PC-3 human prostate cancer cells viability after 24, 48 and 72 h **B.** Different concentrations of noscapine were used to study the effect on 50 nM paclitaxel after 48 h Results were expressed as mean ± SEM. ^*^P<0.05 and ^**^P<0.001. Each point represents the mean of at least three

The effects of paclitaxel and noscapine, alone or in combination on cell viability, was evaluated by MTT assay. The results showed that single and combination treatments of both paclitaxel and noscapine significantly decreased the viability of both LNCaP and PC-3 cells in a dose and time-dependent manner compared to control cells (Figures 1A and B). Differences in sensitivity between two cell types were observed, so that androgen-sensitive LNCaP cells were more sensitive than the hormone-refractory PC-3 cells to both single and combination treatments (*P*<0.05).

In LNCaP cells, paclitaxel induced dose- and time-dependent effect on the viability of this cell line *(P*<0.05). After 24, 48 and 72 h with increasing paclitaxel concentration from 10 to 100 nM, cell viability of LNCaP cells was decreased from 94.2 ± 1.78 to 55.8 ± 6.3, 73.59 ± 0.1 to 42.13 ± 1.57 and 39.4 ± 2.77 to 16.02 ± 0.67% respectively. In contrast, in PC-3, cell viability was decreased from 96.55 ± 1.35 to 59.26 ± 1.26, 82.94 ± 5.24 to 49.2 ± 5.4 and 64.84 ± 2.36 to 39.62 ± 1.6% respectively in response to 24, 48, and 72 h treatments with 10 to 100 nM of paclitaxel (*P*<0.05). 

On the other hand, After 24, 48, and 72 h with increasing noscapine concentration from 10 to 100 µM, cell viability of LNCaP cells was decreased from 96.11 ± 0.62 to 70.7 ± 1.85, 77.86 ± 0.14 to 46.13 ± 1.17, and 40.4 ± 1.6 to 17.78 ± 0.82% respectively (*P*<0.05) (Figure 1A). In contrast, cell viability was decreased from 97.93 ± 0.3 to 77.6 ± 1, 89.1 ± 2.1 to 51.31 ± 3.2, and 80.84 ± 0.1 to 40.18 ± 7.2% respectively in PC-3 cells in response to 24, 48, and 72 h treatments with 10 to 100 µM of concentration of noscapine (*P*<0.05). 

Paclitaxel and noscapine showed IC_50 _of 50 nM and 50 µM against LNCaP cells respectively after 48 h *P*<0.05). Therefore, in other set of experiments, 50 nM paclitaxel was used in the combination treatment with various concentrations of noscapine (10, 25, 50 and 100 µM) and cell viability was evaluated after 48 h. Combination of both agents further decreased the cell viability of LNCaP and PC-3 cells. In these treatments, cell viability percentages in LNCaP cells in 10, 25, 50, and 100 µM noscapine in combination with 50 nM of paclitaxel were 42.32 ± 1.39, 34.26 ± 2.49, 31.29 ± 1.5, and 28.1 ± 3.1, and it were 55 ± 0.5, 47 ± 1.02, 40.62 ± 1.77, and 33.38 ± 1.94% of in PC-3 cells respectively (*P*<0.05) (Figure 1B). 

Data showed that each cell type was signiﬁcantly more sensitive to the combination treatment than to control cells and single treatments indicating a strong synergism between the two drugs. The inhibitory effect on cell viability also was more in LNCaP cells than in PC-3 cells (Figure1 A and B). 

*Induction of apoptosis in LNCaP and PC-3 cells*

**Figure 2:**
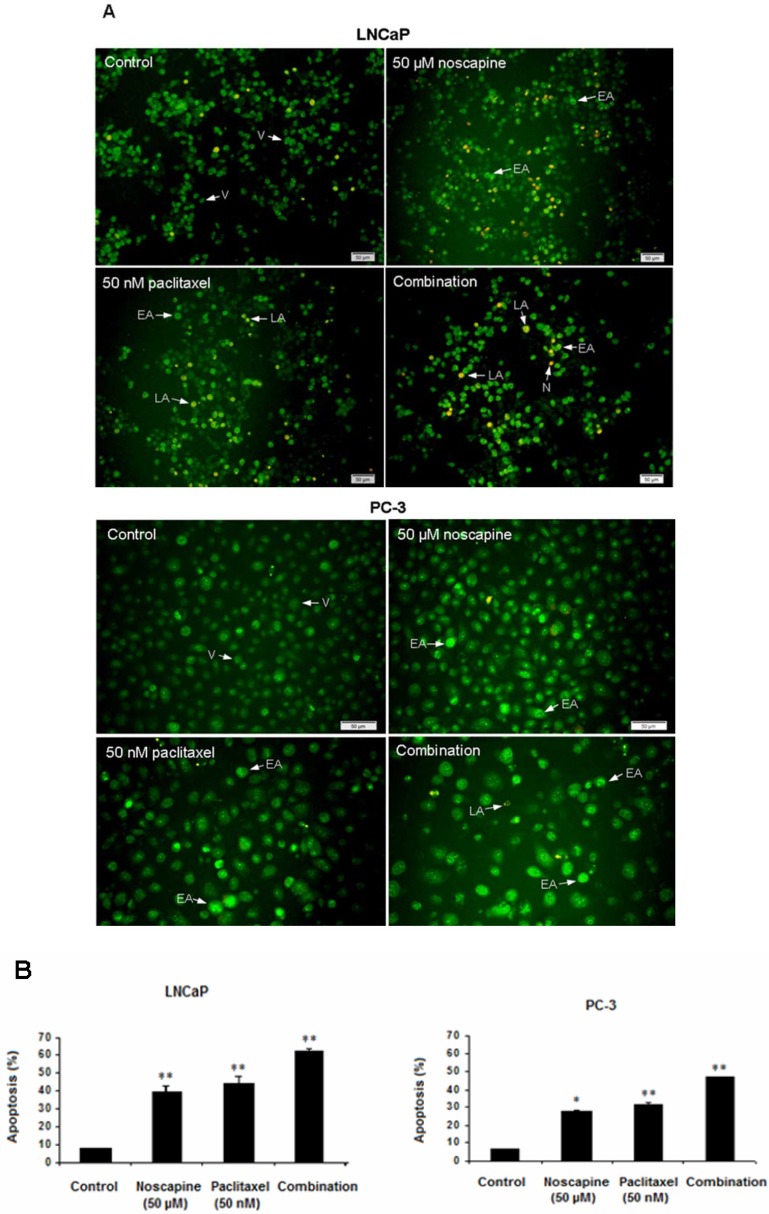
Effect of 50 nM paclitaxel, 50 µM noscapine and their combination on LNCaP and PC-3 human prostate cancer cells apoptosis stained with AO/EB after 48 h A: Fluorescence Micrographs of cells in control, 50 µM noscapine, 50 nM paclitaxel and combination of them after 48 h V: viable, EA: early apoptotic, LA: late apoptotic and N: necrotic cells. Scale bar, 50 µm. **B:** Quantitation of apoptotic LNCaP and PC-3 cells. Results were expressed as mean ± SEM. ^*^P<0.05 and ^**^P<0.001

LNCaP and PC-3 cells undergoing apoptosis following treatment with noscapine (50 µM), paclitaxel (50 nM), and combination compared to untreated cells after 48 h. Apoptosis was demonstrated by staining nuclei with AO/EB staining (Figure 2A). The percentages of treatment-induced apoptosis in LNCaP cells were higher (*P*<0.05) than apoptosis in PC-3 cells in both single and combination treatments. Apoptotic percentages of LNCaP cells were 39.6 ± 3.54 in 50 µM noscapine and 44.5 ± 3.51% in 50 nM paclitaxel (*P*<0.001). While, apoptotic percentages of PC-3 cells were 28.04 ± 0.04 in 50 µM noscapine and 31.7 ± 1.2% in 50 nM paclitaxel (Figure 2B) (*P*<0.05). 

Combination treatments of two drugs were different from single treatments. The data indicated that both cells were more sensitive to combination treatment (*P*<0.05) than to single treatments with differences in apoptotic percentage between LNCaP and PC-3 cells (*P*<0.05). The ratio of combined-treated cells with apoptotic morphology increased dramatically over time and reached about 61.9 ± 1.69 and 47.05 ± 0.34% in treated LNCaP and PC-3 cells respectively after 48 h of treatment (*P*<0.05) compared to controls (Figure 2B), indicating a potential synergism between paclitaxel and noscapine. In addition, AO/EB staining revealed an increased number of apoptotic cells in LNCaP cells (Figure 2A) compared to PC-3 cells (Figure 2B).

*Effect of paclitaxel and noscapine on apoptotic genes expression in LNCaP and PC-3 cells*

The results of this study showed that both of 50 µM noscapine or 50 nM paclitaxel and their combination treatments decreased expression of anti-apoptotic molecule *Bcl-2* and increased expression of pro-apoptotic molecule *Bax *in both LNCaP and PC-3 cells after 48 h. Incubation of LNCaP and PC-3 cells with the combination of paclitaxel and noscapine showed significant decrease in *Bcl-2* mRNA expression to 0.26 ± 0.01 and 0.28 ± 0.06 (*P*<0.001) and showed 2.09 ± 0.06 and 1.54 ± 0.05 fold increased in *Bax* mRNA expression respectively which were significantly different compared to that seen in control groups and treatment with paclitaxel and noscapine alone (*P*<0.001) (Figure 3A, B). A significant increase in *Bax to Bcl-2* ratio of 8.1 ± 0.25 and 4.99 ± 0.5 were observed in LNCaP and PC-3 cells with combined treated of paclitaxel and noscapine respectively (*P*<0.001) (Figure 3B). However, in LNCaP cells, the lower *Bcl-2 *and higher *Bax* and* Bax to Bcl-2* ratio mRNA levels were observed after 48 h of treatment compared to PC-3 cells (*P*<0.001) (Figure 3B)

**Figurer 3:**
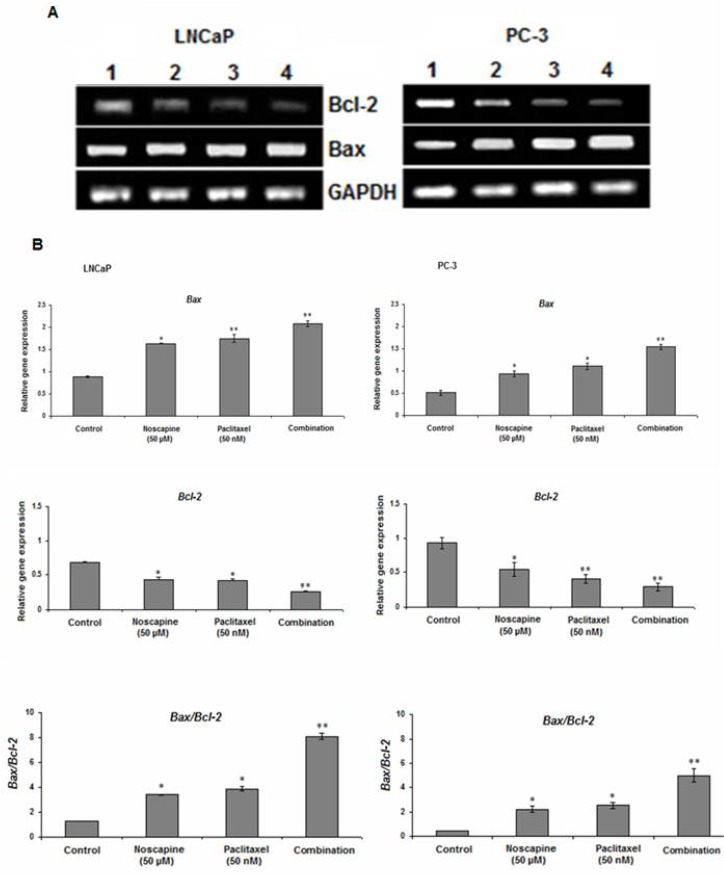
Effect of 50 nM paclitaxel, 50 µM noscapine and their combination on mRNA expression of *Bax *and* Bcl-2* genes of LNCaP and PC-3 human prostate cancer cells using RT-PCR after 48 h **A:** Lane 1: control; Lane 2:50 µM noscapine; Lane 3: 50 nM paclitaxel; Lane 4: Combination. **B:** Relative quantitation of mRNA expression of *Bax* and *Bcl-2* and was quantified against the housekeeping gene (*GA*P*DH*). A significant increase in *Bax/Bcl-2* ratio was observed with LNCaP and PC-3 cells combined treated of paclitaxel and noscapine respectively compared to control cells. Results were expressed as mean ± SEM. ^*^P<0.05 and ^**^P<0.001

**Figure 4:**
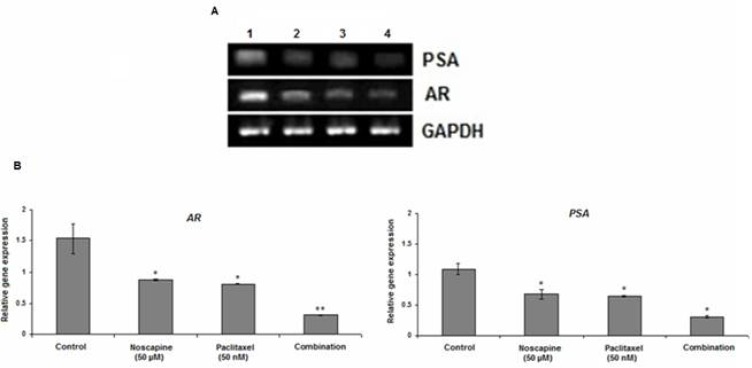
Effect of 50 nM paclitaxel, 50 µM noscapine and their combination on mRNA expression of *AR* and P*SA* in LNCaP cells using RT-PCR after 48 h A**. **Lane 1: control; Lane 2: 50 µM noscapine; Lane3: 50 nM paclitaxel; Lane4: Combination. B**:** Relative quantitation of *AR* and P*SA* mRNA expression against the housekeeping gene (*GA*P*DH)*. Results were expressed as mean ± SEM. ^*^P<0.05 and ^**^P<0.001

*AR* and *PSA* expression 

Our results demonstrated that paclitaxel, noscapine, and their combination inhibited *AR* and *AR*-regulated gene P*SA *expression in LNCaP cells *in-vitro.* The levels of *AR* mRNA expression were considerably reduced in combined-treated group compared to the control (*P*<0.001) (Figure 4A, B). The mRNA *AR* levels in control, 50 µM noscapine, 50 nM paclitaxel and combination treated groups were 1.54 ± 0.23, 0.88 ± 0.02, 0.81 ± 0.01 and 0.31 ± 0.01 while the mRNA P*SA *levels were 1.09 ± 0.09, 0.68 ± 0.8, 0.65 ± 0.02 and 0.31 ± 0.02 respectively (*P*<0.05) (Figure 4B). These results demonstrated that paclitaxel, noscapine, and their combination inhibited LNCaP cells growth probably by a decrease in *AR* and P*SA* mRNA expression. 

## Discussion

In the present study, the synergistic *in-vitro* effect of paclitaxel and noscapine on two human prostate cancer cell lines, LNCaP, and PC-3 was evaluated and MTT assay indicated that paclitaxel, noscapine and their combination induced dose-and time-dependent inhibitory effect on LNCaP and PC-3 cells. However, both cell types were signiﬁcantly more sensitive to combination treatments with paclitaxel and noscapine than single treatments, thus conﬁrming the potential beneﬁcial synergism between two reagents, paclitaxel and noscapine. To our knowledge, this is the first study that demonstrates effectiveness of noscapine in combination with paclitaxel against prostate cancer cells.

According to MTT results, LNCaP cells were more sensitive for either paclitaxel or noscapine and their combination than PC-3 cells. The different response between two cell lines may be due to the involvement of androgen pathway. The taxoid, docetaxel (taxotere) that has a mechanism of action similar to paclitaxel (taxol) regulated many genes including microtubule, apoptosis, and cell cycle-related genes in prostate cancer cell lines, LNCaP and PC-3 cells (3). Also, previous study demonstrated that noscapine is effective in reducing primary tumour growth and lymphatic metastasis of PC-3 human prostate cancer cells in immunodeficient nude mice ^(^26). 

In particular, results of a study by Landen *et al*. demonstrated a clear antitumor advantage in murine melanoma B16LS9 cells and model of established subcutaneous melanoma, receiving noscapine or paclitaxel and the noscapine and paclitaxel combination group compared with untreated animals(15) but the cell lines used were different than in our study.

To study the possible mechanism involved in the anticancer activity of paclitaxel and noscapine combination, induction of apoptosis in LNCaP and PC-3 cells was evaluated. Our results indicated that apoptosis is an important pathway associated with the anticancer activity of paclitaxel and noscapine. According to results obtained using AO/EB staining, pacliaxel and noscapine combination treatments showed significant induction of apoptosis in two cell lines in a synergistic manner in compared to untreated cells and single drug treatments. Other studies showed that paclitaxel-induced programmed cell death (8). Our results were consistent with previous study that illustrated treatment of PC-3 cells with paclitaxel resulting in a dose-dependent inhibition of cell viability and the occurrence of DNA laddering and apoptotic cell death (27). 

Associated with the antitumor activity of these compounds, studies with noscapine and cisplatin combination also showed that their anticancer activity was mediated by induction of apoptosis via intrinsic and extrinsic pathways and inhibition of survival proteins in H 460 lung tumors (28) thus confirming that apoptosis is an important pathway associated with the anticancer activity of these compounds. Also, induction of apoptosis and expression of cleaved caspase-3 was significantly induced *in-vivo* by combination treatment compared to noscapine or doxorubicin alone (29). To elucidate the underlying activity mechanism of paclitaxel or noscapine and their combination, we have evaluated the expression of apoptosis-related genes. We could detect differences in expression of *Bax* and *Bcl-2 *in various treatment groups. These data suggested that single and combination drug treatments induced apoptosis by down regulation of *Bcl-2* and up regulation of *Bax* in both of LNCaP and PC-3 cells, while treatments in a synergistic manner were more effective. 

It has been showed that low doses of paclitaxel enhanced *Bcl-2* phosphorylation and led to its degradation and increasing *Bax* level and induced changes characteristic to apoptosis in human anaplastic cells in thyroid cancer cells (30). Previous reports have indicated that paclitaxel also phosphorylates *Bcl-2* to eliminate its anti-apoptotic effect, which accelerates the release of cytochrome C from mitochondria into the cytosol (31, 32).

Liu *et al*. demonstrated that noscapine increased pro-apototic Bax and decreased anti-apoptotic Bcl-2 proteins that suggested involvement of mitochondrial pathway (33). The present study is therefore in agreement with other studies that demonstrated noscapine alone and combination with doxorubicin induced Bax or decreased Bcl-2 proteins that suggested involvement of mitochondrial pathway and confirmed that apoptosis is an importantpathway (29). 

However, in this study mRNA expression level of apoptosis-related genes in LNCaP cells was higher than PC-3 cells in both combination and single treatments that showed LNCaP is more sensitive to treatment than PC-3 in both combination and single treatments.

Impairing AR activity is a targeting for taxol-based chemotherapy during prostate cancer progression (34). In the present study, we reported that combination of microtubule-targeting chemotherapeutic agent’s paclitaxel and noscapine could interfere with AR activity and inhibited LNCaP cells proliferation accompanied by a reduction of *AR* mRNA expression. These findings suggest that the effects of these drugs may be mediated, at least in part, by inhibition of the AR function, and AR signaling pathway implicated as a therapeutic target.

These observations were in conformity with the results of the previous studies that demonstrated AR plays an important role not only in maintaining the function of the prostate, but also in promoting the development of androgen-dependent and androgen-independent prostate cancers (35, 36). In prostate cancer a preferential expression of membrane ARs has been detected (37) compared with noncancerous cells, correlating to the Gleason’s grade (38), and therefore directly proportional to the aggressiveness of the tumor. 

Also we found that the expression of *PSA*, a well-known *AR* target gene, is also decreased by paclitaxel, noscapine, and their combination in LNCaP cells. In agreement with our results, two published reports have shown that in advanced, refractory prostate cancer, both docetaxel and paclitaxel improve survival, reduce pain, and decrease expression of *AR* levels and P*SA* expression (6, 39) , although with significant side effect (40).

## Conclusions

The present *in-vitro* study indicates that noscapine and paclitaxel combination treatment is effective against LNCaP and PC-3 cells by induction of apoptosis therefore, promising a novel therapeutic strategy to improve the treatment efficiency of prostate cancer therapy.
